# Nitrogen Metabolism and Biomass Production in Forest Trees

**DOI:** 10.3389/fpls.2018.01449

**Published:** 2018-09-28

**Authors:** Francisco M. Cánovas, Rafael A. Cañas, Fernando N. de la Torre, María Belén Pascual, Vanessa Castro-Rodríguez, Concepción Avila

**Affiliations:** Grupo de Biología Molecular y Biotecnología de Plantas, Departamento de Biología Molecular y Bioquímica, Universidad de Málaga, Málaga, Spain

**Keywords:** *Populus*, nitrogen acquisition, nitrogen recycling, glutamine biosynthesis, transgenic trees

## Abstract

Low nitrogen (N) availability is a major limiting factor for tree growth and development. N uptake, assimilation, storage and remobilization are key processes in the economy of this essential nutrient, and its efficient metabolic use largely determines vascular development, tree productivity and biomass production. Recently, advances have been made that improve our knowledge about the molecular regulation of acquisition, assimilation and internal recycling of N in forest trees. In poplar, a model tree widely used for molecular and functional studies, the biosynthesis of glutamine plays a central role in N metabolism, influencing multiple pathways both in primary and secondary metabolism. Moreover, the molecular regulation of glutamine biosynthesis is particularly relevant for accumulation of N reserves during dormancy and in N remobilization that takes place at the onset of the next growing season. The characterization of transgenic poplars overexpressing structural and regulatory genes involved in glutamine biosynthesis has provided insights into how glutamine metabolism may influence the N economy and biomass production in forest trees. Here, a general overview of this research topic is outlined, recent progress are analyzed and challenges for future research are discussed.

## Introduction

Forest trees include a large group of gymnosperm and angiosperm species that play a crucial role in the overall balance of ecosystems. Forest species also have great economic importance in the production of wood, paper pulp, biofuels and a variety of resins and secondary metabolites. The biorefinery and nanotechnology of forest products are emerging areas of industrial interest in Europe within the so-called bioeconomy of the forestry sector (Horizons – Vision 2030 for the European Forest-based Sector^1^). Despite the relevance of forest species from the environmental, economic and social point of view, our knowledge of the mechanisms underlying forest growth, development, and productivity is still limited when compared to crop plants. However, recent developments in genomics and biotechnology are providing new tools to unravel key regulatory processes in fundamental tree biology (Tuskan et al., 2006; De la Torre et al., 2014; Plomion et al., 2016; Tsai et al., 2018).

A sustainable management of forest resources is needed to satisfy the increasing demand of forest-derived products and to preserve natural forest stands. For example, highly productive plantations with increased levels of tree biomass production are necessary to meet the demands of second-generation bioenergy and other forest resources (Hinchee et al., 2009; Allwright and Taylor, 2016). These new forests will require a sustainable use of fertilizers with N as one of the most relevant components. N use efficiency (NUE) is defined in general terms as the amount of plant product per unit of N fertilizer supplied (Good et al., 2004), and trees with improved NUE will be required to enhance the yield of future plantations. Nitrogen acquisition and metabolism are therefore important targets to improve forest biomass production, and key genes involved in N acquisition from soil and assimilation into amino acids have been studied (Cánovas et al., 2007; de la Torre et al., 2014; Castro-Rodríguez et al., 2016a, 2017). In addition, processes of N storage and recycling are particularly relevant in forest species with long life cycles exhibiting seasonal periods of growth and development (Cantón et al., 2005; Minocha et al., 2015). In deciduous trees, most of the leaf N that is present in the stromal and thylakoidal proteins and chlorophylls of green plastids is allocated and stored in the stem during seasonal dormancy; these N reserves are rapidly mobilized to sustain metabolic activities in the next growing season (Babst and Coleman, 2018).

The genus *Populus* (poplars) include a variety of tree species with fast growth in temperate habitats. In fact, poplars are widely used for biomass production and considered to be one of the most important bioenergy crops (Ye et al., 2011; Allwright and Taylor, 2016). In addition, *Populus* has become a model tree due to its favorable characteristics for experimental analyses and the advances made during the last 15 years in its structural and functional genomics (Jansson and Douglas, 2007; Douglas, 2017). In this article, the functional genomics of N metabolism in *Populus* is reviewed. The relevance of these studies to enhance biomass production is highlighted. Finally, potential avenues for future research on this topic are discussed.

## N Acquisition and Metabolism in Poplar

Poplars are able to acquire inorganic N forms from soil, such as NH_4_^+^ and NO_3_^-^, and its relative preference will depend on the soil pH (Rennenberg et al., 2010). The genome of *Populus trichocarpa* contains an abundant repertoire of genes encoding low- and high-affinity transporters involved in N uptake and allocation. NO_3_^-^ uptake is mediated by a large family of transporters consisting of 68 *PtNPF* genes encoding nitrate and peptide transporters and a smaller family of 11 *PtNRT2/NRT3* genes also encoding nitrate transport systems (Bai et al., 2013). NH_4_^+^ transporters (AMT) are encoded by 22 genes distributed in two separate subfamilies (*AMT1* and *AMT2*) (Couturier et al., 2007; Wu et al., 2015; Calabrese et al., 2017). Interestingly, the number of AMT2 members in poplar is much higher than in *Arabidopsis*, suggesting differences in the way that these plants incorporate and transport NH_4_^+^ ions (Castro-Rodríguez et al., 2017; Gojon, 2017). The specific expression patterns of these genes strongly suggest that they play non-overlapping roles in N acquisition and intercellular transport (Castro-Rodríguez et al., 2017).

Regardless of the source of inorganic N that is taken up by the roots, NH_4_^+^ is the ultimate N form to be assimilated into amino acids. The main step for N entry in plant metabolism is catalyzed by the enzyme glutamine synthetase (GS; EC 6.3.1.2) and it involves the ATP-dependent condensation of NH_4_^+^ and glutamate for the biosynthesis of glutamine. Unlike that found in other plant species, GS is encoded by a duplicated gene family in *Populus* consisting of 4 groups of genes, 3 of which code for GS isoforms of cytosolic localization (GS1.1, GS1.2, and GS1.3) and one group that codes for a plastid-located isoform (GS2). Duplicated *GS* genes display similar structures with well conserved regulatory regions and intron-exon boundaries. However, they are dispersed in the poplar genome and distributed on separate chromosomes (Castro-Rodríguez et al., 2011). Functional analyses of recombinant duplicates revealed that they exhibit similar molecular and kinetic properties and therefore are functionally equivalent enzymes (Castro-Rodríguez et al., 2015). The specific spatial and seasonal patterns of expression of GS genes support non-overlapping roles in poplar N metabolism (Castro-Rodríguez et al., 2011).

**FIGURE 1:**
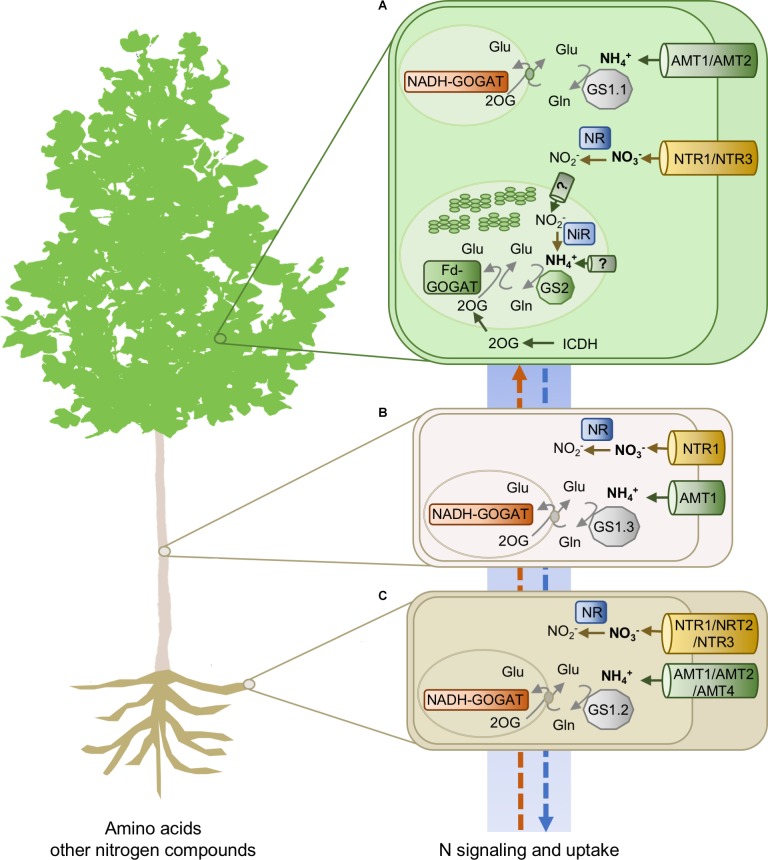
Nitrogen transporters and enzymes involved in N acquisition and metabolism in poplar. **(A)** leaves, **(B)** stem, and **(C)** roots. AMT1, ammonium transporter 1; AMT2, ammonium transporter 2; NRT1, nitrate transporter 1 (NPF); NRT2, nitrate transporter 2; NRT3, nitrate transporter 3; GS1.1, cytosolic glutamine synthetase, isoform 1; GS1.2, cytosolic glutamine synthetase, isoform 2; GS1.3, cytosolic glutamine synthetase, isoform 3; GS2, glutamine synthetase, chloroplastic isoform; Fd-GOGAT, ferredoxin-dependent glutamate synthase; NADH-GOGAT, NADH-dependent glutamate synthase; ICDH, cytosolic isocitrate dehydrogenase; NiR, nitrite reductase; NR, nitrate reductase.

*In situ* hybridization and laser capture microdissection of the whole GS family disclosed that the expression of duplicates is confined to specific cell-types, confirming and extending previous findings (Castro-Rodríguez et al., 2015). Expression studies supported a relevant role of the GS1.1 isoforms in N metabolism of photosynthetic cells in coordination with the role of the chloroplast-located GS2, for example, in the reassimilation of NH_4_^+^ released during photorespiration (Betti et al., 2006). In contrast, the role of the GS1.2 enzymes could be related to N mobilization during seasonal N recycling at the onset of dormancy and with senescence associated with pathogen attack. Finally, the expression patterns of the GS1.3 isoforms are consistent with playing an essential role in the biosynthesis of glutamine for N transport (Cánovas et al., 2007) and reassimilation of NH_4_^+^ released in the metabolism of phenylalanine during wood formation (Craven-Bartle et al., 2013). Interestingly, genes encoding glutamate synthases (GOGAT, EC 1.4.7.1, and EC 1.4.1.14) and cytosolic isocitrate dehydrogenase (ICDH, EC 1.1.1.42) are also duplicated in poplar. The main players in N uptake and metabolism are shown in **Figure 1**. Gene IDs are listed in **Supplementary Table S1**.

Enzyme redundancy in glutamine biosynthesis occurs in particular cell types of poplar. Paralogous genes may have been retained in the poplar genome to increase the amount of enzyme because their expression is restricted to specific cell-types, and the accumulation of a GS isoform could contribute to maintaining the homeostasis of the N metabolism in a particular cell-type. Glutamine biosynthesis is at the crossroad of many metabolic pathways, and according to the above hypothesis, functions associated with glutamine-derived metabolic products would be enhanced in specialized tissues such as meristems, photosynthetic parenchyma, xylem, and phloem of vascular bundles. The availability of enhanced levels of organic N in the form of glutamine could have boosted the growth and vigor of these plants, favoring adaptability to changes in environmental conditions and colonization of new habitats.

## Gene Functional Analysis in Transgenic Trees

**FIGURE 2:**
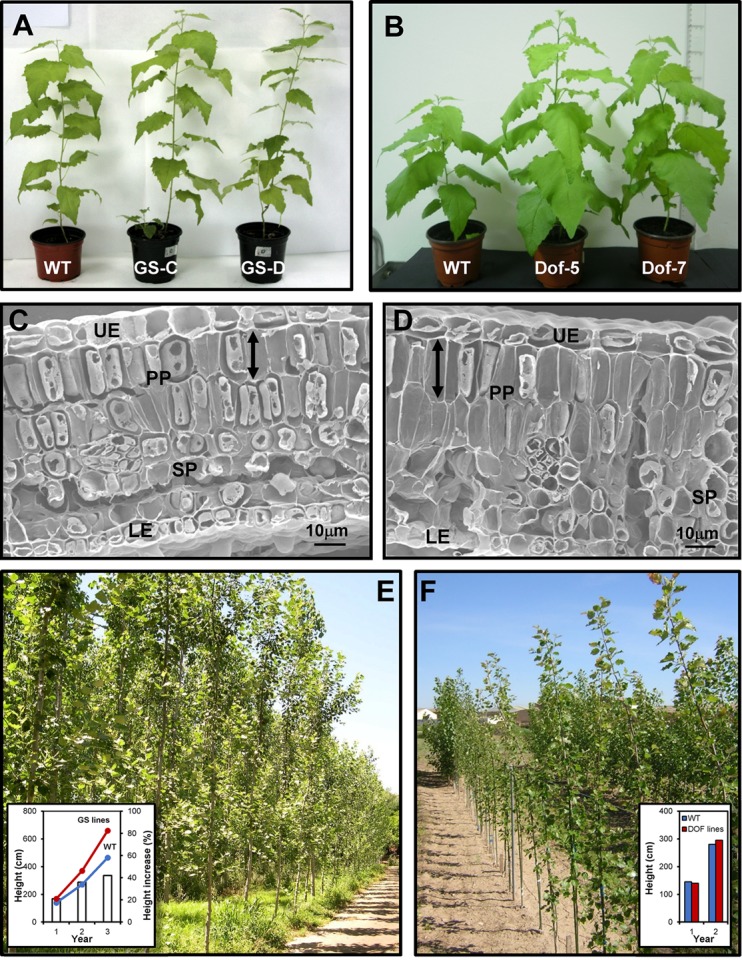
Photographs of transgenics in the laboratory and field trials (*Populus tremula x P. alba*). **(A)** Wild-type and GS transgenic lines. **(B)** Wild-type and Dof transgenic lines. Scanning electron micrographs of transversal sections of young leaves from wild-type **(C)** and transgenic **(D)** poplars overexpressing ICDH; UE, upper epidermis; LE, lower epidermis; SP, spongy parenchyma; PP, palisade parenchyma. Palisade mesophyll cells were longer in the transgenics than in control plants (arrows). **(E)** GS transgenic trees growing in natural conditions. **(F)** Field trial of Dof transgenic trees. Histograms of growth are shown in the insets.

Classical breeding has been widely used for tree improvement but new developments in genomics and biotechnology can accelerate the process. In regard to N nutrition, the above results point to GS as a key enzyme in N metabolism; however, it is important to elucidate which GS isoform contributes to a major extent to poplar growth and biomass production. Transgenic hybrid poplars (*P. tremula x P. alba*) overexpressing a cytosolic GS of pine exhibited enhanced growth and increased levels of proteins and chlorophylls (Fu et al., 2003; **Figure 2A**). Furthermore, the observed phenotype as a consequence of transgene expression was related to the correct assembly of GS1 subunits in the cytosol of photosynthetic cells. Further characterization showed that GS transgenics had a better NUE (Man et al., 2005; Castro-Rodríguez et al., 2017) and enhanced tolerance to abiotic stress (El-Khatib et al., 2004; Pascual et al., 2008; Shestibratov et al., 2010; Molina-Rueda and Kirby, 2015). A similar approach, overexpressing *GS1*, has also been used to improve NUE in birch species (Lebedev et al., 2017).

Enhanced growth of GS transgenics was associated with increases in the transcript and protein levels of anthranilate synthase, the enzyme catalyzing the biosynthesis of tryptophan, a precursor of auxin biosynthesis (Man et al., 2011). These findings highlight the paramount importance of GS1 in poplar growth and biomass production, which has also been demonstrated in herbaceous plant models such as maize (Hirel et al., 2007) and rice (Tabuchi et al., 2007). In contrast, chloroplastic GS plays a well-established role in reassimilation of NH4^+^ released in the photorespiratory pathway as determined by characterization of photorespiratory mutants lacking GS2 (Blackwell et al., 1987; Betti et al., 2006).

The ability of a regulatory gene to influence growth and biomass production has also been tested in poplar. Dof factors are regulators of N metabolism and potential targets to enhance N assimilation and plant growth (Rueda-López et al., 2008; Tsujimoto-Inui et al., 2009; Wang Y. et al., 2013). The transcription factor Dof5 that regulates GS1 isoforms in maritime pine (Rueda-López et al., 2008) was overexpressed in hybrid poplars (**Figure 2B**). In comparison to untransformed controls, young transgenic plants exhibited enhanced growth, an increased capacity for inorganic N uptake, and accumulated significantly more carbohydrates and lignin (Rueda-López et al., 2017).

The assimilation of NH_4_^+^ into amino acids by the GS/GOGAT pathway also requires the provision of carbon skeletons in the form of 2-oxoglutarate (2-OG) (Hodges, 2002). The role of ICDH, a key enzyme in the provision of 2-OG, has been investigated in hybrid poplar and overexpression of *ICDH* causes an alteration in vascular development (Pascual et al., 2018). Transgenic trees with higher levels of ICDH also displayed increased expression of *GS1.3* and other genes associated with vascular differentiation. Phenotypic characterization of the transgenic plants showed increased growth in height, longer internodes and enhanced development in young leaves and the apical region of the stem (**Figures 2C,D**). *ICDH* overexpression altered the contents of organic acids including citrate, malate and 2-OG, and the levels of glutamate and γ-aminobutyric acid. These results show that the provision of carbon skeletons for NH_4_^+^ assimilation and glutamine biosynthesis is a key metabolic process for growth and vascular development in poplar.

Field trials of genetically modified trees are extremely important to assess transgene behavior under natural conditions and potential risks of transgenes spreading before commercialization. A field trial of independently transformed lines expressing GS1 was established, and the performance of these transgenic lines was studied in natural conditions over 3 years (Jing et al., 2004; **Figure 2E**). The transgene was stably expressed in the field resulting in enhanced vegetative growth of transgenic poplars reaching average heights that were 41% greater than non-transformed controls (Jing et al., 2004; Cánovas et al., 2006). These results likely reflect a higher capacity of transgenic trees for N remobilization and N recycling, resulting in a better exploitation of nutrient resources. Interestingly, analysis of wood samples from these 3-year-old trees revealed alterations in cell wall characteristics resulting in improved attributes for pulp and paper production (Coleman et al., 2012). GS1 transgenics also tolerate high levels of NO_3_^-^ supply, exhibiting greater NUE and accumulating increased biomass, particularly enriched in cellulose in the above-ground part of the plant (Castro-Rodríguez et al., 2016b). These results are consistent with an efficient N allocation and metabolism in the transgenics. Transcriptomic analysis revealed that transgenic trees are able to reprogramme the transcriptome in response to N excess by differential expression of a greater number of genes than untransformed plants. The above findings strongly support the potential use of these genetically modified trees for phytoremediation of NO_3_^-^ pollution with enhanced production of biomass and cellulose for bioenergy applications.

The performance of transgenic trees overexpressing Dof5 was also studied in a field trial during two growing seasons (**Figure 2D**). Interestingly, these transgenic lines showed attenuated growth and no modification of carbon or N metabolism when growing under natural conditions. As the expression of the transgene was stable during the period of study the observed differences in the performance of transgenic trees were attributed to the low levels of N nutrients available in the soil (Rueda-López et al., 2017). These findings reinforce the importance of field studies and indicate that the manipulation of structural rather than regulatory genes has been more effective for increasing biomass production and forest productivity. **Figure 2** illustrates the phenotypes of genetically modified poplars growing under controlled and natural conditions.

## Perspectives and Future Developments

Recent advances made in model and crop plants highlight the importance of NO_3_^-^ and NH_4_^+^ transporters as key components of NUE. Consequently, manipulation of N acquisition and intercellular transport should be addressed to explore potential benefits in growth and productivity. In rice plants, overexpression of NO_3_^-^ transporters such as OsNRT1.1B (Hu et al., 2015) and OsNRT2.3a (Fan et al., 2017) led to an accumulation of more biomass and increased yield. The identification of potential orthologs of these genes in poplar and subsequent functional analysis will deserve special attention to increasing tree productivity. Additional efforts are also needed to characterize amino acid transporters, particularly those involved in mechanisms of N allocation and recycling (Babst and Coleman, 2018). A strict coordination between N transporters and GS isoforms needs to exist to sustain the glutamine flux that is necessary for the biosynthesis of all nitrogenous compounds during poplar growth and development. A comparative analysis of gene expression in poplar showed co-expression profiles for several *AMT1*, *AMT2*, and *GS1* genes in young leaves, mature leaves and stems (Castro-Rodríguez et al., 2017). The coordinated function of N transporters and GS1 in different poplar tissues need to be investigated in future studies. Interestingly, Zhang et al. (2018) reported that under low nitrogen, the excess of carbon is redirected to the biosynthesis of aromatic amino acids and lignin, resulting in improved NUE that could be of practical value in terms of biomass production. Deciphering of regulatory networks involved in the response of roots to nitrogen availability is also deserving special attention (Wei et al., 2013; Dash et al., 2015; Luo et al., 2015).

As previously discussed, the manipulation of glutamine biosynthesis is a reasonable strategy to improve NUE and biomass production in poplar. According to the currently available data, the overproduction of GS1 isoforms in particular cell-types is largely beneficial; however, further studies are necessary to fully understand how the increase in glutamine biosynthesis influences tree growth and biomass production. For example, the specific contribution of the GS1 duplicates, GS1.1, GS1.2, and GS1.3, should be explored by performing functional studies using classic transformation strategies, or alternatively, by using the powerful CRISPR-Cas9 technology (Zhou et al., 2015). A recent study reported that overexpression of poplar *GS1.2* in tobacco altered secondary cell wall and fiber characteristics and accelerated auxin biosynthesis (Lu et al., 2018), but unfortunately, we still do not know what the impact of *GS1.2* manipulation in poplar may be. Of particular interest will be to specifically elucidate how the GS1.1 duplicates are associated with photosynthetic primary and/or secondary NH_4_^+^ assimilation in mature leaves. The impact of GS1 overproduction can be further studied with refined approaches using tissue-specific and inducible promoters (Filichkin et al., 2006; Wang L. et al., 2013).

Nevertheless, the existence of intrinsic regulatory mechanisms *in planta* cannot be ruled out. GS1 expression driven by the constitutive 35S promoter is potentially modulated in photosynthetic tissues through the interaction with a microRNA leading to improved biomass production (Fu et al., 2012). Furthermore, the co-transformation of GS1 transgenics with cellulase genes driven by inducible promoters could facilitate processing of feedstocks for bioenergy applications. Another challenge for the future is the identification and molecular dissection of QTLs potentially associated with GS1/glutamine biosynthesis. QTLs for biomass production under nitrogen limitation and excess have been mapped in poplar but candidate genes of N metabolism were not identified (Novaes et al., 2009). In contrast, interactions have been shown between genes involved in glutamine and glutamate metabolism and QTLs associated to yield traits in maize (Hirel et al., 2007) and rice (Yamaya et al., 2002).

It is worth mentioning that the observed effects of GS1 transgene expression are explained by the altered expression of other genes involved in primary and secondary metabolism. Significant changes in the leaf transcriptome were observed when growing trees at high NO_3_^-^ levels with a high number of genes differentially expressed, including those involved in photosynthesis, cell wall formation and phenylpropanoid biosynthesis (Castro-Rodríguez et al., 2016b). In turn, the upregulation of transcription factors strongly suggests that chromatin organization differs in transgenics and wild-type plants, particularly in the response of trees to N availability. Genome-wide identification of regions containing targeted genes involved in the nutritional responses can be achieved by conducting comparative ChiP-Seq analysis in poplar (Liu et al., 2015). A recent genomic resource will facilitate this task, the whole genome-assembly of the *P. tremula* x *P. alba* clone INRA 717-1B4 that is used as a tree model in transgenic experiments (Mader et al., 2016). New knowledge derived from these studies and/or those derived from the molecular dissection of QTLs will facilitate the identification of genes linking GS1/glutamine biosynthesis and biomass production. Using gene capture approaches (Seoane-Zonjic et al., 2016), structural variability in these genes can be analyzed in poplar genotypes with a contrasted ability for biomass production. In fact, a recent study has confirmed substantial structural variation in the poplar pan-genome (Pinosio et al., 2016).

## Conclusion

A combination of functional genomic approaches, including transgenic and gene editing technology, chromatin analysis, and systematic identification of genome regions involved in NUE, will facilitate the exploration of the molecular basis of how N metabolism influences biomass production in forest trees.

## Author Contributions

FC and CA conceived and wrote the manuscript. RC, FdlT, MP, and VC-R made additional contributions and edited the manuscript. RC and VC-R composed the figures.

## Conflict of Interest Statement

The authors declare that the research was conducted in the absence of any commercial or financial relationships that could be construed as a potential conflict of interest.

## Footnotes

1 http://www.forestplatform.org/

## Supplementary Material

TABLE S1 Gene IDs and accession numbers for N transporters and enzymes involved in N acquisition and metabolism in poplar.
