# Supplementary material for: Nitrogen Metabolism and Biomass Production in Forest Trees
Source: Front Plant Sci. 2018 Sep 28;9:1449. doi: 10.3389/fpls.2018.01449 (PMC6172323; doi:10.3389/fpls.2018.01449)
Supplement: TABLE S1 — Gene IDs and accession numbers for N transporters and enzymes involved in N acquisition and metabolism in poplar. [file Table_1.DOCX]

**Supplementary Table S1.** Gene IDs and accession numbers for N transporters and enzymes involved in N acquisition and metabolism in poplar.

Nitrate and ammonium transporters in leaves involves multiples players such as PtNRT1.9C, PtNRT1a3, PtNRT1c2B, PtNRT1c5F, PtNRT1d8A, PtNRT1d8C, PtNRT3.1A, as well as PtrAMT1;1, PtrAMT1;3, PtrAMT1;6, PtrAMT2;1. GS isoenzymes involved during ammonium assimilation are PtGS1.1-831163, PtGS1.1-710678 in the cytosol and PtGS2-725763 and PtGS2-820914 in the chloroplast. In stems, PtNRT1.2A, PtNRT1.5C, and PtNRT1c2A are highly expressed. The major isoform involved in ammonium transporter is PtrAMT1;1 and GS members expressed in the stems are PtGS1.3-834185 and PtGS1.3-827781. PtNRT1a1A, PtNRT1c5E, PtNRT1d5B, PtNRT2.4A, and PtNRT3.1B are involved in nitrate assimilation in roots. The most important root-specific ammonium transporter is PtrAMT1;2 but also PtrAMT1;5, PtrAMT2;2 and PtrAMT4;5; PtGS1.2-819912 and PtGS1.2-716066 are responsible for ammonium assimilation.

| **Genes ID** | **Accession number** |
| --- | --- |
| PtNRT1.9C | Potri.001G351200 |
| PtNRT1a3 | Potri.001G068600 |
| PtNRT1c2B | Potri.016G103500 |
| PtNRT1c5F | Potri.013G106600 |
| PtNRT1d8A | Potri.010G126300 |
| PtNRT1d8C | Potri.016G032000 |
| PtNRT3.1A | Potri.012G089300 |
| PtrAMT1;1 | Poptr1_1: 804848 |
| PtrAMT1;3 | Poptr1_1: 565016 |
| PtrAMT1;6 | Poptr1_1: 804509 |
| PtrAMT2;1 | Poptr1_1: 802015 |
| PtGS1.1-831163 | Potri.004G085400.1 |
| PtGS1.1-710678 | Potri.017G131100.1 |
| PtGS2-725763 | Potri.010G029100.1 |
| PtGS2-820914 | Potri.008G200100.1 |
| PtNRT1.2A | Potri.012G070700 |
| PtNRT1.5C | Potri.014G179400 |
| PtNRT1c2A | Potri.006G092000 |
| PtrAMT1;1 | Poptr1_1: 804848 |
| PtGS1.3-834185 | Potri.012G043900.1 |
| PtGS1.3-827781 | Potri.015G034700.1 |
| PtNRT1a1A | Potri.003G000800 |
| PtNRT1c5E | Potri.T150700 |
| PtNRT1d5B | Potri.018G040500 |
| PtNRT2.4A | Potri.009G008600 |
| PtNRT3.1B | Potri.015G0851000 |
| PtrAMT1;2 | Poptr1_1: 665333 |
| PtrAMT1;5 | Poptr1_1: 645545 |
| PtrAMT2;2 | Poptr1_1: 808726 |
| PtrAMT4;5 | Poptr1_1: 424130 |
| PtGS1.2-819912 | - |
| PtGS1.2-716066 | Potri.005G093200.1 |
